# *Candida* Bloodstream Infections Among Persons Who Inject Drugs — Denver Metropolitan Area, Colorado, 2017–2018

**DOI:** 10.15585/mmwr.mm6812a3

**Published:** 2019-03-29

**Authors:** Devra M. Barter, Helen L. Johnston, Sabrina R. Williams, Sharon V. Tsay, Snigdha Vallabhaneni, Wendy M. Bamberg

**Affiliations:** ^1^Colorado Department of Public Health and Environment; ^2^Division of Foodborne, Waterborne, and Environmental Diseases, National Center for Emerging and Zoonotic Infectious Diseases, CDC.

Candidemia, a bloodstream infection caused by *Candida* species, is typically considered a health care–associated infection, with known risk factors including the presence of a central venous catheter, receipt of total parenteral nutrition or broad-spectrum antibiotics, recent abdominal surgery, admission to an intensive care unit, and prolonged hospitalization (*1*,*2*). Injection drug use (IDU) is not a common risk factor for candidemia; however, in the context of the ongoing opioid epidemic and corresponding IDU increases, IDU has been reported as an increasingly common condition associated with candidemia (*3*) and methicillin-resistant *Staphylococcus aureus* bacteremia (*4*). Little is known about the epidemiology of candidemia among persons who inject drugs. The Colorado Department of Public Health and Environment (CDPHE) conducts population-based surveillance for candidemia in the five-county Denver metropolitan area, encompassing 2.7 million persons, through CDC’s Emerging Infections Program (EIP). As part of candidemia surveillance, CDPHE collected demographic, clinical, and IDU behavior information for persons with *Candida*-positive blood cultures during May 2017–August 2018. Among 203 candidemia cases reported, 23 (11%) occurred in 22 patients with a history of IDU in the year preceding their candidemia episode. Ten (43%) of the 23 cases were considered community-onset infections, and four (17%) cases were considered community-onset infections with recent health care exposures. Seven (32%) of the 22 patients had disseminated candidiasis with end-organ dysfunctions; four (18%) died during their hospitalization. In-hospital IDU was reported among six (27%) patients, revealing that IDU can be a risk factor in the hospital setting as well as in the community. In addition to community interventions, opportunities to intervene during health care encounters to decrease IDU and unsafe injection practices might prevent infections, including candidemia, among persons who inject drugs.

Candidemia surveillance in the five-county Denver metropolitan area began in May 2017. Because candidemia is a reportable condition in the Denver metropolitan area, all surveillance area laboratories report *Candida*-positive blood cultures to CDPHE. As part of EIP surveillance, a case is defined as a blood culture positive for *Candida* spp. in a surveillance area resident; a recurrent case is defined as a new *Candida*-positive blood culture >30 days after the initial positive blood culture in the same patient. Cases were classified by patient epidemiologic exposures. Community-onset infections were defined as *Candida*-positive blood culture collected <3 days after hospital admission with no previous health care exposures (i.e., overnight hospitalizations, surgeries, long-term care, or long term acute care admissions in the previous 90 days and no central lines in place in the 2 days prior to culture collection). Health care–associated, community-onset infections were defined as a *Candida*-positive blood culture collected <3 days after hospital admission with previous health care exposures in the 90 days before the culture collection date. Hospital-onset infections were defined as blood cultures collected ≥3 days into the patient’s hospitalization. Medical record reviews were performed to gather demographic and clinical information, including history of IDU, for all patients using a standardized case report form.

During the first 6 months of the surveillance program, CDPHE observed that approximately one in 10 cases of candidemia occurred in patients who had a documented history of IDU, and the majority of their *Candida*-positive blood cultures were collected on the day of hospital admission or shortly thereafter. This finding was unexpected given that candidemia typically occurs in severely ill, hospitalized patients (*1*,*2*). CDPHE and CDC conducted an epidemiologic investigation to describe candidemia among persons who inject drugs and identify potential interventions for prevention. For each case occurring in a person with documented IDU, medical records were reviewed to collect information on health care exposures, evidence of disseminated infections, coinfections, and drug use and associated practices before the *Candida*-positive cultures.

Among 203 candidemia cases reported during May 2017–August 2018, 23 (11%) were identified in 22 patients with IDU in the past year; one patient had recurrent candidemia. Among these 22 patients, the average age was 37 years (range = 21–59 years), and 14 (64%) were women (Table). Eighteen (82%) of the patients were white, and four (18%) were Hispanic or Latino. Eleven (50%) patients had experienced homelessness or lived in transitional housing before the candidemia episode. Among the 22 candidemia patients with IDU, 10 (45%) had hepatitis C infection, including one who also had chronic hepatitis B infection and one who also had human immunodeficiency virus (HIV) infection. Other comorbidities included chronic lung disease; neurologic conditions such as seizures, epilepsy, or neuropathy; diabetes; alcohol abuse; and smoking tobacco during the preceding year.

**TABLE:** Characteristics of 22 patients with candidemia and a history of injection drug use — Denver metropolitan area, Colorado, May 2017–August 2018

Characteristic	No. (%)
**Mean age, yrs (range)**	37 (21–59)
**Sex**	
Women	14 (64)
Men	8 (36)
**Race**	
White	18 (82)
Asian	1 (5)
American Indian/Alaska Native	1 (5)
Unknown	2 (9)
**Ethnicity**	
Not Hispanic or Latino	18 (82)
Hispanic or Latino	4 (18)
**Comorbidities**	
Hepatitis C, chronic	9 (41)
Hepatitis C, acute	1 (5)
Hepatitis B, chronic	1 (5)
Human immunodeficiency virus infection	1 (5)
Chronic lung disease*	4 (18)
Neurologic condition^†^	8 (36)
Diabetes	5 (23)
Alcohol abuse	4 (18)
Smoking tobacco	18 (82)
**Experiencing homelessness/Transitional housing**	11 (50)
**Outcome**	
Died	4 (18)
Left against medical advice	3 (14)
Survived	15 (68)
***Candida* species in incident blood culture^§^**	
*Candida glabrata*	8 (35)
*Candida albicans*	6 (26)
*Candida parapsilosis*	3 (13)
*Candida dubliniensis*	2 (9)
*Candida lusitaniae*	1 (4)
*Candida lypolitica*	1 (4)
*Candida rugosa*	1 (4)
*C. albicans and Candida famata*	1 (4)
**Epidemiologic class^§^**	
Community-onset^¶^	10 (43)
Health care–associated, community-onset**	4 (17)
Hospital-onset^††^	9 (39)
**Hospitalization for candidemia (median days, range)^§^**	23 (10, 1–139)
**Risk factors before *Candida*-positive blood culture^§^**	
Admission to an intensive care unit^§§^	6 (26)
Recent abdominal surgery	0
Presence of a central venous catheter^¶¶^	11 (48)
Receipt of broad-spectrum antibiotics^§§^	20 (87)
Receipt of total parenteral nutrition	0

* Includes chronic pulmonary diseases such as chronic obstructive pulmonary disease, emphysema, chronic bronchitis, bronchiectasis, interstitial lung disease, and asthma.

^†^ Includes epilepsy/seizure/seizure disorder and neuropathy.

^§^ Among 23 candidemia cases, which included one recurrent case.

**^¶^** Positive blood culture <3 days after hospital admission without prior health care exposures in the previous 90 days.

** Positive blood culture collected <3 days after hospital admission with prior health care exposure in the previous 90 days.

^††^ Positive blood culture collected ≥3 days into hospital admission.

^§§^ 14 days before the *Candida*-positive blood culture.

**^¶¶^** On the day of, or in the 2 calendar days before the *Candida*-positive blood culture.

Three patients left the hospital against medical advice, possibly without completing treatment for candidemia. Three additional patients had left against medical advice from at least one other medical encounter in the 6 months before their candidemia episode. Four (18%) patients died in the hospital 1–17 days after the *Candida*-positive blood culture, although whether candidemia was the direct cause of death was unknown.

Among the 23 infections in these 22 patients, the most common *Candida* species identified were *Candida glabrata*, *Candida albicans*, and *Candida parapsilosis* (Table). Ten (43%) of the 23 cases were identified as community-onset infections. Four (17%) cases were identified within 1 day of the patient’s hospital admission or during a previous emergency department (ED) visit or hospitalization, after which the patient returned for treatment; these patients also had other health care exposures and were classified as health care–associated, community-onset infections. Nine (39%) cases were classified as hospital-onset infections. Among the nine patients with hospital-onset candidemia, the median interval from hospital admission to collection of the *Candida*-positive blood culture was 17 days (range = 4–107 days). Among all 23 candidemia cases, the median length of the candidemia-associated hospitalization was 10 days (range = 1–139 days).

In the 6 months before developing candidemia, the 22 patients had a mean of three previous inpatient or ED visits (range = 0–10). Including the admission for candidemia, the most common reasons for admission or ED visit were conditions related to drug use (i.e., dependence or withdrawal); nonspecific pain; mental or behavioral disorders; and infections and associated complications, including bacteremia, osteomyelitis, and sepsis.

Fifteen (68%) of the 22 patients had a blood culture yielding another organism (most commonly *Staphylococcus aureus*) either during the candidemia hospitalization or in the 6 months preceding the candidemia episode. In 10 (45%) patients, at least one other organism was identified in the same blood culture set as the one that yielded *Candida* spp. These included *Staphylococcus aureus,* coagulase negative *Staphylococcus, Stenotrophomonas maltophilia, Pseudomonas fluorescens, Serratia marcescens, Enterobacter asburiae, Comamonas acidovorans, Pantoea* spp., viridans *Streptococcus,* and *Mucorales* spp. Seven (32%) patients had disseminated candidiasis with end-organ dysfunctions, including endophthalmitis (one), septic emboli (one), osteomyelitis (three), and abscesses of the pelvis, psoas muscle, and upper mediastinum (three).

Drugs documented in the medical record or identified in urine testing in the 6 months before the candidemia episode included opioids (18 patients; 82%), methamphetamines (16; 73%), cannabinoids (seven; 32%), cocaine (six; 27%), benzodiazepines (four; 18%), ecstasy (MDMA) (one), and barbiturates (one). Two patients (9%) experienced “cotton fever,” an illness characterized by rapid fever onset immediately following the injection of drugs filtered through cotton (*5*), in the 6 months before the candidemia hospitalization; four patients (18%) were reported to have engaged in unsafe injection practices, including using old syringes, cotton, filters, and dirty needles.

Six (27%) patients were observed injecting or attempting to inject drugs, including illicit drugs and pain medications that were not prescribed to them, while hospitalized. In addition, illicit drugs and drug paraphernalia, including syringes, spoons, and lighters, were found in four of these six patients’ rooms.

## Discussion

Surveillance for candidemia in the Denver metropolitan area during 2017–2018 found that approximately one in 10 patients with candidemia had recent IDU. Patients with candidemia who had a history of IDU had high prevalences of IDU-associated conditions, including hepatitis C infection, homelessness, and disseminated candidiasis with end-organ dysfunction, increasing candidemia-associated morbidity; four of these patients died during their candidemia hospitalization.

Fourteen of the 22 patients in this analysis presumably became infected with *Candida* outside the hospital setting, likely related to IDU. In addition, the positive polymicrobial blood cultures in nearly half of the patients indicate that unsafe injection practices likely are prevalent, putting persons who inject drugs at risk for candidemia and other infections with bacteria, HIV, hepatitis C virus, and hepatitis B virus. Nine patients became infected in the hospital setting, likely because they either continued to inject drugs while hospitalized (six were observed injecting or attempting to inject drugs) or they had more typical health care–associated risk factors for candidemia.

The findings in this report are subject to at least two limitations. First, a relatively small number of patients with candidemia and a history of IDU were identified in the Denver-metropolitan area during the study period; therefore, results of this analysis might not be generalizable to other geographic areas. Second, because IDU behaviors were identified by reviewing patient medical records, some patients with a history of IDU might have been missed if IDU practices were not documented. Similarly, information such as type of drugs used, frequency of use, and unsafe injection practices might not have been documented in the medical record.

This surveillance program identified IDU as a previously underrecognized risk factor for candidemia in Colorado. Usual candidemia prevention efforts, including antibiotic stewardship, catheter care, and antifungal prophylaxis, primarily occur in the health care setting to mitigate health care–associated risk factors. Prevention of candidemia and other infections in persons who inject drugs requires both health care and community-based interventions such as education about and resources for reducing IDU, increasing safe injection practices (e.g., cleaning the injection site, using sterile water in drug preparation, and avoiding shared injection equipment*), and initiation of medication-assisted treatment programs for persons injecting opioids. 

Patients in this analysis were found to have had frequent health care encounters, including ED visits and inpatient admissions, in the 6 months preceding their candidemia episode. These health care encounters provide opportunities for targeted prevention efforts in addition to community-based interventions. Given the current opioid epidemic, it is important to monitor trends in drug use and IDU-related infections and to implement prevention interventions.

**Summary**

**What is already known about this topic?**

Candidemia is typically considered a health care–associated infection, but injection drug use (IDU) has emerged as an increasingly common condition associated with candidemia.

**What is added by this report?**

Among 203 candidemia cases in the Denver metropolitan area during May 2017–September 2018, 23 (11%) occurred in 22 patients who had a recent history of IDU. Many had disseminated infections with end organ dysfunction, and onset occurred both inside and outside the hospital setting. Six of the patients were observed injecting or attempting to inject drugs while hospitalized.

**What are the implications for public health practice?**

Opportunities to intervene during health care encounters to decrease IDU and unsafe injection practices might prevent infections, including candidemia. Preventing candidemia among persons who inject drugs requires both community-based and health care–based interventions.
